# Global Evidence of the Unimodal Response of Ecosystem Respiration to Soil Moisture

**DOI:** 10.1002/advs.202509753

**Published:** 2025-10-30

**Authors:** Jinlong Peng, Shudi Xie, Jiwang Tang, Jiaqiang Liao, Chen Chen, Chuanlian Sun, Yiheng Wang, Qingping Zhou, Guirui Yu, Shuli Niu

**Affiliations:** ^1^ Key Laboratory of Ecosystem Network Observation and Modeling, Institute of Geographic Sciences and Natural Resources Research Chinese Academy of Sciences Beijing 100101 China; ^2^ College of Resources and Environment University of Chinese Academy of Sciences Beijing 100049 China; ^3^ State Key Laboratory of Urban and Regional Ecology, Research Center for Eco‐Environmental Sciences Chinese Academy of Sciences Beijing 100085 China; ^4^ Institute of Qinghai‐Tibetan Plateau Southwest Minzu University Chengdu 610041 China

**Keywords:** carbon, ecosystem, optimality, respiration, soil moisture

## Abstract

Ecosystem respiration (ER) is the largest flux of carbon from land to the atmosphere and is strongly influenced by soil moisture. Nevertheless, the response of ER to soil moisture remains poorly understood. Here, the ER‐soil moisture response curves at 135 sites from the global FLUXNET are analyzed. In contrast to the typically assumed monotonic increase in respiratory carbon emissions with increasing soil moisture derived from land surface models, the study shows that 106 sites exhibit a unimodal soil moisture response with a peak in ER at an apparent optimum soil moisture (SMER opt), implying a prevalent inhibitory effect of soil moisture excess on ER. Among the 12 biotic and abiotic variables examined, the SMER opt is mostly related to local water availability, with drier sites showing lower SMER opt than wetter sites, possibly reflecting water adaptation of ER. This adaptation is further supported by a field experiment that exclusively manipulates water and holds other factors constant, showing a downward shift in SMER opt after long‐term water deficit. These results reveal the large variation of SMER opt and the water adaptation of ER, providing novel insights for understanding and forecasting carbon‐climate feedbacks.

## Graphical abstract

Using global eddy covariance observations and a field experiment, this paper provides compelling evidence for the widespread unimodal relationship between soil moisture and ecosystem respiration and its water adaptation. Such knowledge facilitates understanding of soil moisture in affecting respiratory carbon emissions and has the potential to improve the predictive power of global carbon cycling in the context of climate change.

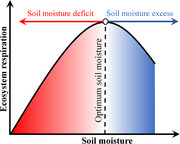

## 1 Introduction

Terrestrial ecosystems release around ten times more carbon dioxide (CO_2_) through respiration than is emitted by anthropogenic burning of fossil fuels.^[^ ^1^, ^2^, ^3^, ^4^ ^]^ This is known as ecosystem respiration (ER), the cumulation of microbial or heterotrophic respiration and plant or autotrophic respiration, which represents the largest CO_2_ flux from land to atmosphere.^[^ ^4^, ^5^, ^6^ ^]^ It has been proposed that soil moisture critically influences respiratory CO_2_ emissions,^[^ ^7^, ^8^, ^9^ ^]^ but accurately characterizing the effect of soil moisture on ER is notoriously challenging in land surface models,^[^ ^10^, ^11^, ^12^ ^]^ leading to huge uncertainties in assessing carbon‐climate feedbacks.^[^ ^13^, ^14^, ^15^ ^]^ A comprehensive understanding of the response of ER to soil moisture is increasingly crucial.^[^ ^7^, ^11^ ^]^

**Figure 1:**
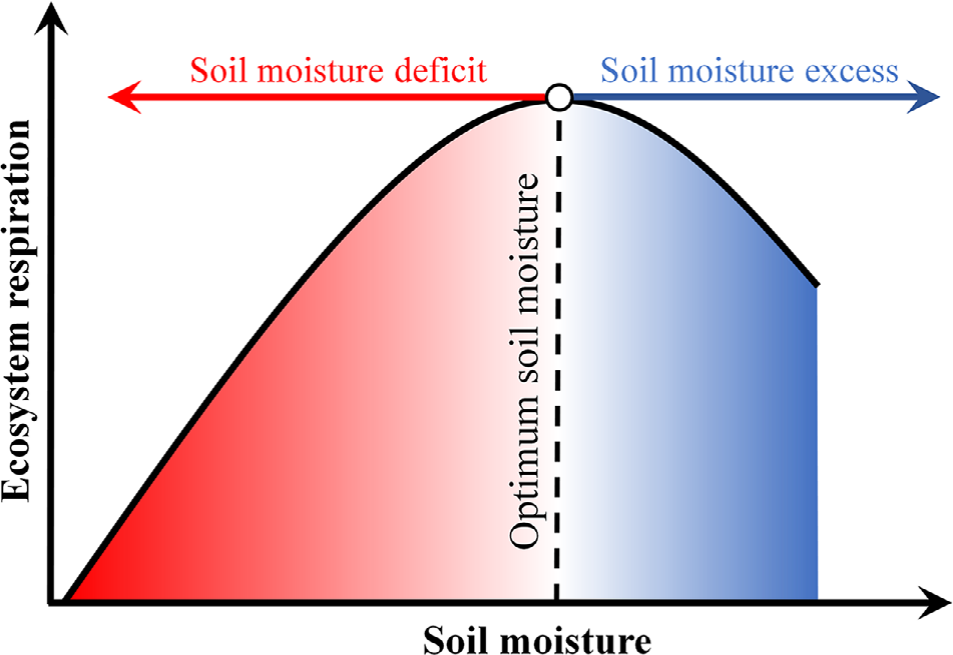
Conceptual framework of relationship between soil moisture and ecosystem respiration (ER). The vertical black dotted line indicates optimum soil moisture beyond which the effect of soil moisture on ER shifts from positive to negative (SMER opt). The red arrow represents soil moisture deficit (i.e., soil moisture below SMER opt), and the bule arrow represents soil moisture excess (i.e., soil moisture above SMER opt).

In general, a rise in soil moisture can stimulate ER, as water is a dominant limiting factor for respiratory processes.^[^ ^8^, ^9^, ^16^ ^]^ Accordingly, most current models (e.g., community land model 5.0) usually account for the stress of soil moisture deficit on respiration by incorporating an empirical monotonic function, with a consensus that respiration decreases at lower values of soil moisture down to a point where respiration is minimal.^[^ ^10^, ^11^, ^13^ ^]^ This respiratory suppression caused by soil moisture deficit is expected to be more pronounced under warmer conditions,^[^ ^17^ ^]^ as elevated temperatures typically exacerbate drought by intensifying atmospheric vapor pressure deficit, thereby imposing greater water stress on plants and microorganisms.^[^ ^18^ ^]^ However, more and more recent studies have indicated an inhibitory effect of soil moisture excess on both autotrophic and heterotrophic respiration,^[^ ^19^, ^20^, ^21^, ^22^, ^23^, ^24^ ^]^ even in ecosystems that are typically water‐deficient.^[^ ^9^ ^]^ This inhibition is suggested to be the consequence of a progressive soil anoxia and a short supply of respiratory substrates, which impairs respiratory activity of plants and microbes.^[^ ^8^, ^11^, ^20^ ^]^ In this context, the ER can be expected to follow a unimodal dynamic of increase, peak and then decrease in response to increasing soil moisture, with a threshold, which we have termed the apparent optimum soil moisture (SMER opt), corresponding to the maximum ER (**Figure** **1**). Despite this ER‐soil moisture framework being conceptually established,^[^ ^4^, ^11^, ^21^ ^]^ the quantification of SMER opt to delineate transition of soil moisture effects on ER from positive to negative remains limited. So far, at the global scale, there has been no observation‐based assessment of SMER opt, although a few studies have shown examples of a unimodal relationship between soil moisture and ER at site scale.^[^ ^21^, ^25^, ^26^ ^]^ Even less is known about the mechanisms that control SMER opt. Recognizing these is pertinent to build confidence in simulating global carbon cycle, given that the majority of ecosystems are projected to experience a significant variability of soil moisture.^[^ ^27^, ^28^, ^29^ ^]^

In this study, to quantify SMER opt directly, we used eddy covariance observations from the FLUXNET dataset and analyzed the response curves of ER to soil moisture at globally distributed 135 sites. The analysis showed that ER at 106 sites exhibited unimodal response curves with increasing soil moisture, and SMER opt exists widely with large variability among sites. We then examined a serial of climatic, edaphic, and vegetation factors that potentially explain the variation in SMER opt. Among these factors, local water availability exhibited the strongest explanatory power, with drier sites showing lower SMER opt than wetter sites, possibly reflecting water adaptation of ER. This adaptation is further supported by a field experiment that solely manipulates water and keeps other factors constant, which showed a downward shift in SMER opt after long‐term water deficit. Overall, we addressed two specific research questions: i) whether SMER opt exists widely and ii) what are the mechanisms that control SMER opt variation around the globe.

## 2 Results

### 2.1 Existence of SMER opt

**Figure 2:**
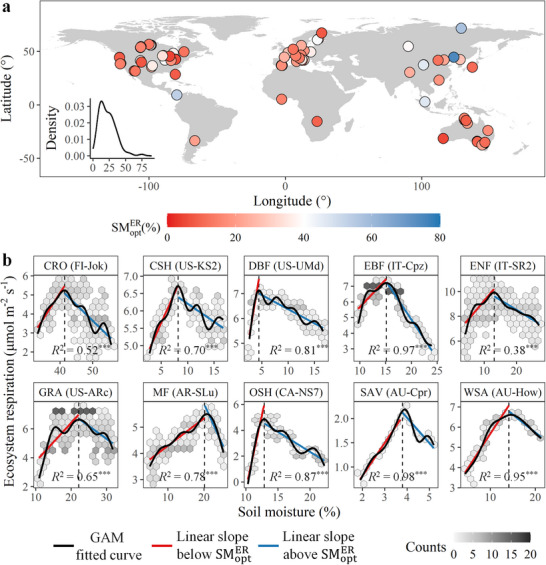
Distribution of optimum soil moisture for ecosystem respiration (SMER opt) derived from FLUXNET sites. a) Location of the 106 sites used in this study with detected SMER opt. The inset plot shows the distribution of SMER opt values. b) Density plots showing the response of ecosystem respiration to soil moisture at the 10 sites from 10 different vegetation types. The black solid curve indicates the fitting of generalized additive model (GAM), and the vertical black dotted line represents the detected SMER opt. The *R* ^2^ value shows the result of GAM. The red and blue lines indicate the fitting of linear regression on both sides of the SMER opt. Site name is shown in parentheses after each vegetation type. The vegetation types are as follows: CRO, croplands; CSH, closed shrublands; DBF, deciduous broadleaf forests; EBF, evergreen broadleaf forests; ENF, evergreen needle‐leaf forests; GRA, grasslands; MF, mixed forests; OSH, open shrublands; SAV, savanna; WSA, woody savanna. ^***^, *p* ≤ 0.001; ^**^, *p* ≤ 0.01; ^*^, *p* ≤ 0.05.

We detected the presence of SMER opt at 106 sites, covering large areas and most vegetation types from a total of 135 sites in our dataset (see Experimental Section; **Figure** **2**a; Figure S1 and Table S1, Supporting Information). Quantitative analysis showed that ER first increased, peaked, and then declined with increasing soil moisture, and data from 10 sites representing different vegetation types were plotted to illustrate this unimodal relationship (Figure 2b). Across the 106 sites, SMER opt ranged from 3.9 to 74.5%, with a median of 20.5%. Considering that heat and light are potentially correlated or co‐vary with soil moisture, we further tested the robustness of the observed existence of SMER opt against the impacts of incoming shortwave radiation, air temperature, and vapor pressure deficit. Specifically, we first conducted partial Spearman's correlation analysis to estimate the strength of the non‐linear relationship between soil moisture and ER while controlling for incoming shortwave radiation, air temperature, and vapor pressure deficit (i.e., partial Spearman's r). We then estimated the strength of the non‐linear relationship between soil moisture and ER without controlling for any factors (i.e., Spearman's r). Finally, we tested the relationship between partial Spearman's r and Spearman's r. The results showed a strong linear relationship between them (Figure S2, Supporting Information), and the linear slope was not significantly different from 1 (95% confidence interval, 0.86 to 1.16). This suggests that incoming shortwave radiation, air temperature, and vapor pressure deficit had a negligible effect on the non‐linear relationship between soil moisture and ER. Thus, the observed transition from an increase to a decrease in ER with increasing soil moisture (i.e., the presence of SMER opt) was not likely caused by these confounding factors. Consistently, we found similar results when we used explainable machine learning to determine SMER opt by controlling for incoming shortwave radiation, air temperature, and vapor pressure deficit (see Experimental Section; Figures S3 and S4, Supporting Information).

**Figure 3:**
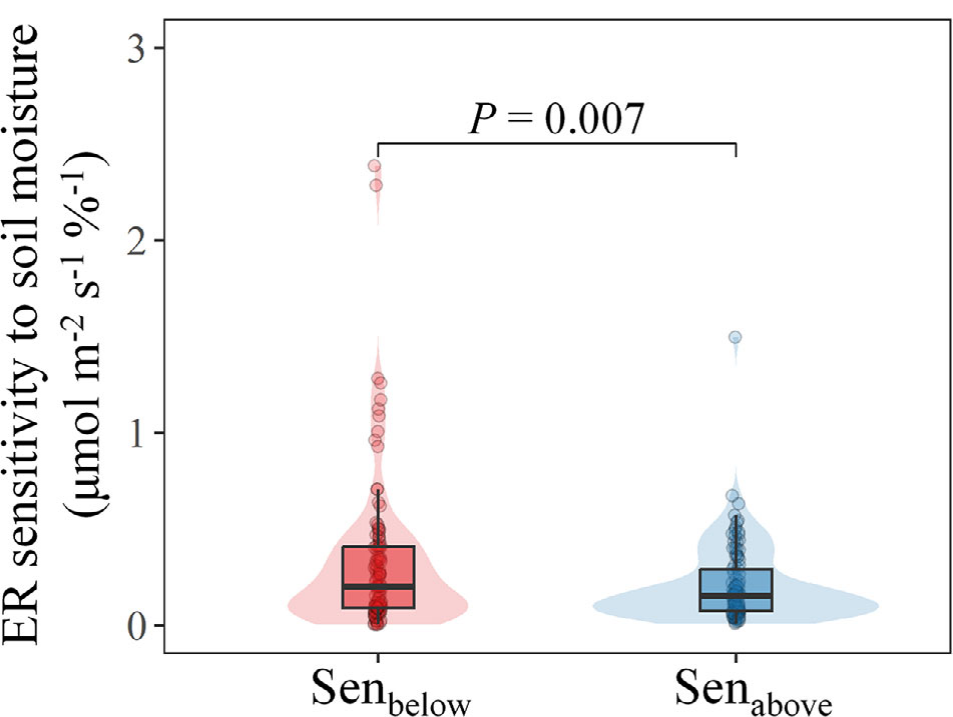
Sensitivity of ecosystem respiration (ER) to soil moisture at both sides of the optimum soil moisture for ER (SMER opt). Sen_below_ and Sen_above_ refer to the absolute values of ER‐soil moisture linear slopes below and above the SMER opt, respectively. Violin plot shows the distribution of Sen_below_ and Sen_above_. Box plot lines represent the interquartile range and median, whereas the whiskers represent 1.5 times the interquartile range. The *P* value shows the result of paired Wilcoxon test.

At each site with detected SMER opt, we further used linear regression to estimate the ER sensitivity to soil moisture below (Sen_below_) and above SMER opt (Sen_above_), which were represented by the absolute values of ER‐soil moisture slopes on both sides of the SMER opt (see Supporting Information). The Sen_below_ ranged from 0.01 to 2.39 µmol m^−2^ s^−1^ %^−1^, with a median of 0.20 µmol m^−2^ s^−1^ %^−1^, and the Sen_above_ ranged from 0.01 to 1.49 µmol m^−2^ s^−1^ %^−1^, with a median of 0.15 µmol m^−2^ s^−1^ %^−1^ (**Figure** **3**). The paired Wilcoxon test showed that across the 106 sites, the mean of Sen_below_ was significantly higher than that of Sen_above_ (see Supporting Information). However, it should be noted that there were still 46 sites exhibiting higher Sen_above_ than Sen_below_ (Figure S5, Supporting Information), which accounted for 43.4% of all sites. This suggests that the depressing effect of soil moisture excess on ER can sometimes be comparable to or even greater than that of soil moisture deficit.

### 2.2 Drivers of the Spatial Variability of SMER opt

**Figure 4:**
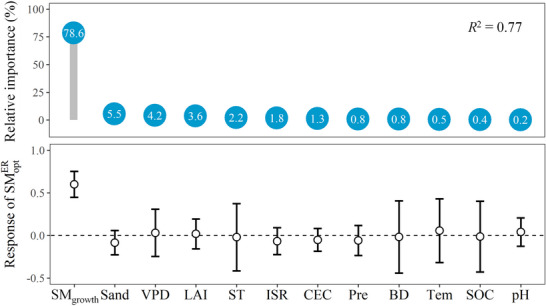
Moderators for optimum soil moisture for ecosystem respiration (SMER opt). The relative importance of moderators was estimated by relative weight analysis (RWA), with numbers in the circles representing the rescaled relative weight. The response of SMER opt to moderators was estimated by ridge regression. The circles and bars represent standardized coefficients and 95% confidence intervals (CI), respectively, and the standardized coefficient was significant when the 95% CI did not overlap with zero (horizontal black dotted line). SM_growth_, growth soil moisture (%); Sand, soil sand fraction (%); VPD, vapor pressure deficit (hPa); LAI, leaf area index; ST, soil temperature (°C); ISR, incoming shortwave radiation (W m^−2^); CEC, soil cation exchange capacity (cmol kg^−1^); Pre, total precipitation (mm); BD, soil bulk density (kg dm^−3^); Tem, air temperature (°C); SOC, soil organic carbon (%); pH, soil pH.

**Figure 5:**
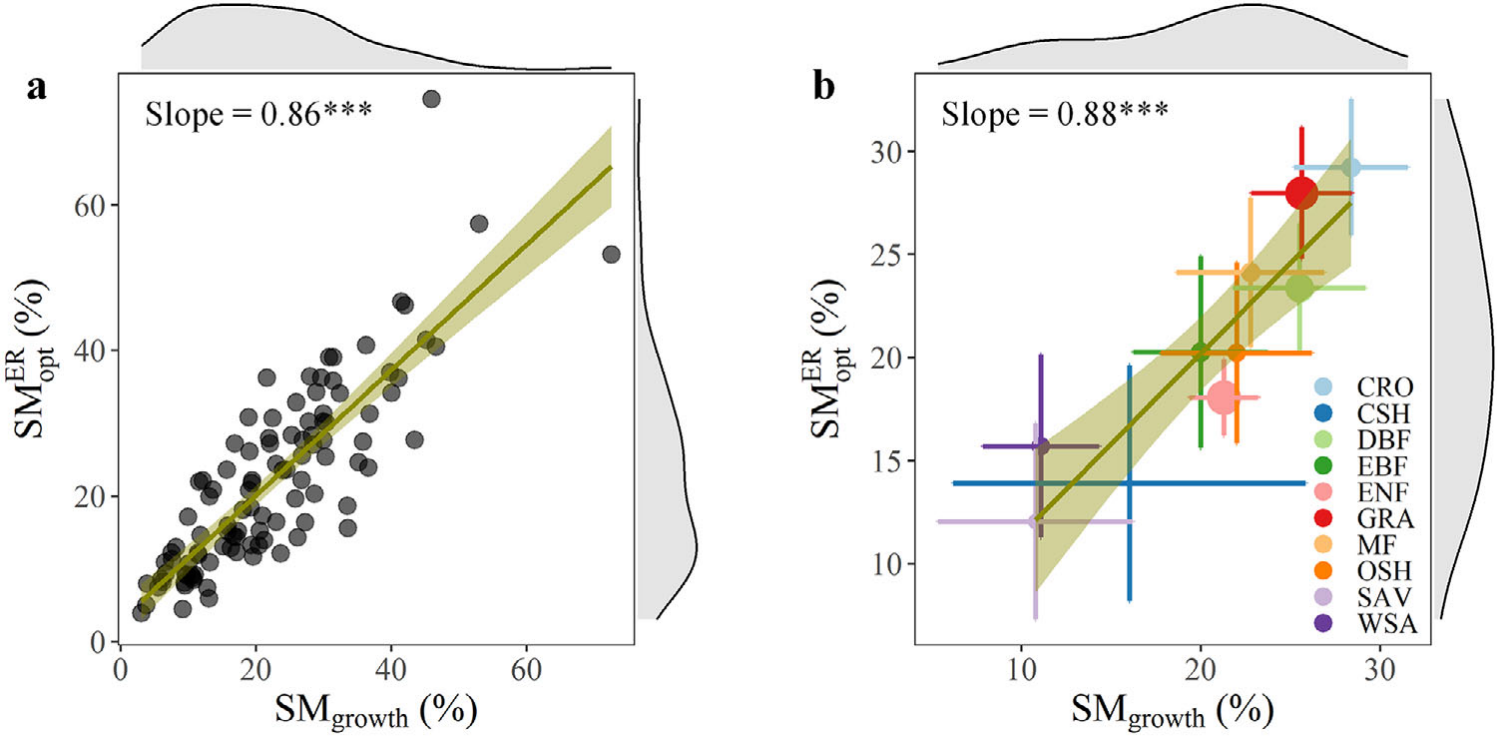
Relationship between optimum soil moisture for ecosystem respiration (SMER opt) and growth soil moisture (SM_growth_). a) Linear regression of SM_growth_ to SMER opt across 106 sites. b) Linear regression of SM_growth_ to SMER opt across 10 vegetation types. The top and right margins show the distribution of SM_growth_ and SMER opt, respectively. Point with error bar indicates the mean with standard error. Point size is proportional to the number of sites in each vegetation type. CRO, croplands, n = 7; CSH, closed shrublands, n = 2; DBF, deciduous broadleaf forests, n = 17; EBF, evergreen broadleaf forests, n = 7; ENF, evergreen needle‐leaf forests, n = 26; GRA, grasslands, n = 24; MF, mixed forests, n = 7; OSH, open shrublands, n = 6; SAV, savanna, n = 4; WSA, woody savanna, n = 6. Solid line and shaded area indicate the linear regression fit and its 95% confidence interval respectively. ^***^, *p* ≤ 0.001; ^**^, *p* ≤ 0.01; ^*^, *p* ≤ 0.05.

We analyzed the relative importance of 12 biotic and abiotic factors on the spatial variability of SMER opt across sites using relative weight analysis (see Experimental Section; **Figure** **4**). The results showed that these predictors together explained 77% of the spatial variability of SMER opt across sites, with the growth soil moisture (SM_growth_, measured by mean soil moisture during the growing season; see Experimental Section) being the most important. Consistently, ridge regression further confirmed that SMER opt was largely determined by SM_growth_, while other factors including incoming shortwave radiation, air temperature, total precipitation, vapor pressure deficit, leaf areas index, soil temperature, soil organic carbon, soil cation exchange capacity, soil pH, soil bulk density, and soil sand fraction did not significantly influence SMER opt, although some of them were significant in the linear regression (Figure S6, Supporting Information). Further analysis showed that the SMER opt shifted upwards with an increase in SM_growth_ across sites and biomes, with slopes of 0.86 and 0.88, respectively (**Figure** **5**). Taken together, these results showed that wetter ecosystems tend to operate at the higher SMER opt, while drier ecosystems tend to operate at the lower SMER opt.

## 3 Discussion

In land‐surface models, the effect of soil moisture on respiratory CO_2_ emissions is typically represented by a monotonic scalar ranging from 0 to 1, with 1 representing maximum respiration without any water limitation.^[^ ^10^, ^11^, ^13^ ^]^ Nevertheless, in this study, based on global eddy covariance observations, we detected the widespread SMER opt over large geographical regions ranging from 121.6° W to 148.5° E and 37.4° S to 71.6° N. We found that among the 12 biotic and abiotic variables examined, the SMER opt was mostly related to the local growth soil moisture (SM_growth_), with drier ecosystems exhibiting lower SMER opt than wetter ecosystems.

The widespread SMER opt indicates that maximum ER often occurs at moderate soil moisture, while minimum ER occurs at both extreme soil moisture deficit and excess. The previously overlooked depressing effect of soil moisture excess was sometimes comparable to or even greater than that of soil moisture deficit (Figure 3; Figure S5, Supporting Information). It is thus clear that the monotonic response curves used in most current models are not the best function to represent the relationship between soil moisture and respiratory CO_2_ emissions. Notably, the depression of soil moisture excess on ER does not necessarily equate to an increase in ecosystem carbon sinks, as the decreased carbohydrate investment in respiration would produce less basic energy (e.g., adenosine triphosphate) needed by organisms to support their survival and growth, which in turn may inhibit ecosystem photosynthesis.^[^ ^30^, ^31^ ^]^ For instance, there is usually a compensatory increase in plant photosynthesis following stimulation of respiration and carbohydrate depletion.^[^ ^32^, ^33^ ^]^

The presence of SMER opt is probably due to multiple mechanisms related to physical, physiological, and biochemical processes, such as the progressive oxygen limitation under soil moisture excess, which suppresses the respiratory activity of plant roots and aerobic microorganisms.^[^ ^11^, ^19^, ^34^, ^35^ ^]^ This is because oxygen diffuses much more slowly through water than through air in the soil pore space.^[^ ^36^ ^]^ Soil moisture excess can also induce nutrient leaching that exacerbates the nutrient limitation of ecosystem photosynthesis,^[^ ^37^, ^38^, ^39^, ^40^, ^41^ ^]^ thereby reducing the supply of substrates for plant and microbial respiration.^[^ ^20^, ^31^, ^42^ ^]^ This was well supported by recent studies, which showed the widespread depression of excessive soil water on gross primary productivity in terrestrial ecosystems.^[^ ^21^, ^23^, ^43^ ^]^ Nevertheless, due to the lack of corresponding datasets in eddy covariance observations, we cannot further test these processes and mechanisms, particularly the role of plant and microbial respiration responses to soil moisture in determining the SMER opt.

Furthermore, we also acknowledge that the eddy covariance‐observed transition from an increase to a decrease in ER with increasing soil moisture may be caused not only by soil moisture itself, but also by other potentially confounding environmental factors, especially heat and light.^[^ ^44^, ^45^, ^46^ ^]^ However, we found that the non‐linear relationship between soil moisture and ER remained similar after controlling for incoming shortwave radiation, vapor pressure deficit, and air temperature (Figure S2, Supporting Information), indicating that the widespread presence of SMER opt is unlikely to be caused by these confounding factors. This was further corroborated by the explainable machine learning, which showed that when controlling for incoming shortwave radiation, vapor pressure deficit, and air temperature, ER still exhibited unimodal response curves with increasing soil moisture at most sites (Figures S3 and S4, Supporting Information).

The close relationship between SM_growth_ and SMER opt may reflect the adaptation of ecosystems to local hydroclimatic conditions. In particular, when SM_growth_ declines, ecosystems down‐regulate their SMER opt to mitigate drought‐induced ER injury. The explanation is that a decline in SM_growth_ only begins to inhibit ER when SM_growth_ falls below SMER opt, so a lower SMER opt implies a higher drought threshold. The maintenance of respiration has important implications for ecosystems to cope with drought, as it provides the basic energy for plants and microbes to access nutrients (e.g., microbial mineralization and active transport of ions by plants) and to synthesize substances that maintain osmotic equilibrium (e.g., proline).^[^ ^6^, ^30^, ^47^ ^]^ In contrast, as SM_growth_ increases, the up‐regulated SMER opt indicates a greater potential for ER rises. To the best of our knowledge, this study is among the first to reveal the adaptation of ER to SM_growth_ changes.

We speculate that two potential mechanisms may be responsible for this observed higher SMER opt under higher SM_growth_, including respiratory substrates and organism activity. Specifically, under wetter climate conditions, there are typically richer carbohydrates and more active plants and microorganisms,^[^ ^16^, ^48^ ^]^ possibly requiring more water to reach maximum ER. These hypotheses are well supported by a long‐term water manipulation experiment in alpine meadow of Qinghai‐Tibet Plateau (see Supplementary Methods and Results), where we found significant positive relationships between SMER opt and aboveground plant biomass and microbial biomass carbon (Figures S7–S9, Supporting Information). High aboveground plant biomass facilitates the synthesis of photosynthetic substrates for respiration, as photosynthesis takes place primarily in the aboveground part of plants,^[^ ^30^ ^]^ and high microbial biomass carbon usually indicates large energy source and strong metabolic activity for microbial respiration.^[^ ^49^, ^50^ ^]^ Nevertheless, issue remains whether the mechanisms proposed by our experiment are fully responsible for the observed adaptation of ER to SM_growth_ changes at global scale. Further investigation based on global network of control experimental studies (e.g., International Drought Experiment^[^ ^51^ ^]^) would provide insights into this issue.

Our study sheds light on understanding and predicting carbon‐climate feedbacks in at least two ways. First, the widespread SMER opt indicates that both soil moisture deficit and excess can inhibit respiratory CO_2_ emissions. Unfortunately, most current models only consider the stress of soil moisture deficit on respiration by using an empirical monotonic function (i.e., a continuous increase in ER with increasing soil moisture),^[^ ^10^, ^11^, ^13^ ^]^ without representing the inhibitory effect of soil moisture excess. This could lead to a large overestimation of ER in relatively wet conditions, as our results showed that the previously overlooked inhibitory effect of soil moisture excess on respiratory CO_2_ emissions can sometimes be comparable to or even greater than that of soil moisture deficit (Figure S5, Supporting Information). Therefore, we suggest that developing alternative models that account for the presence of SMER opt has the potential to more realistically predict global carbon dynamics under climate change. That being said, the lack of detection of SMER opt also occurred in some sites (see Supplementary Results). It may be because these sites did not experience a sufficiently wide range of soil moisture during the observation period, and thus the ER peaks were unable to be detected clearly (Figure S10, Supporting Information). This deserves further investigation when longer‐term observations become available.

Second, the adaptation of ER to SM_growth_ has important implications for ecosystems in the face of future climate change, and studies that do not account for this adaptation are likely to misestimate the trends in respiratory CO_2_ emissions (e.g.,^[^ ^12^, ^52^ ^]^). It should be noted that the adaptation was observed not only by the eddy covariance measurements, but also by the field experiment that showed a down‐regulation of SMER opt after long‐term drought treatment (Figure S7, Supporting Information), demonstrating the causal effect of SM_growth_ on SMER opt. The respiratory substrates and organism activity would be critical in reflecting the adaptation of ER to SM_growth_ change. As shown in the field experiment, ecosystems down‐regulated their SMER opt after long‐term soil water deficit mainly by decreasing aboveground plant biomass and microbial biomass carbon (Figures S8 and S9, Supporting Information). That being said, we only used the surface soil moisture data due to the limited measurements of deep soil moisture. Further investigation of the SMER opt and the water adaptation of ER is therefore warranted in future studies that account for deeper soil moisture, particularly in forest ecosystems where root systems can extend beyond depths of one‐meter and have great potential to access groundwater.^[^ ^53^ ^]^

In summary, we show that contrary to the generally accepted notion of a monotonic relationship between respiratory CO_2_ emissions and soil moisture as derived from land surface models, ER follows a unimodal dynamic of increase, peak and then decrease in response to rising soil moisture, with SMER opt existing widely across different biomes around the globe. The widespread existence of SMER opt is also supported by explainable machine learning, which can effectively distinguish the impact of soil moisture on ER from that of other potentially confounding factors, going beyond mere correlation‐based analysis. We further uncover the controlling factors for the variation of SMER opt, and find that drier ecosystems tend to operate with lower SMER opt than wetter ecosystems, reflecting ecosystem adaptation to local hydroclimatic conditions. Given the importance of ER in the terrestrial carbon cycle, our study provides new insights for models to improve the predictive ability of the carbon cycle under climate change.

## 4 Experimental Section

### FLUXNET Data

FLUXNET was a global network of micrometeorological sites that provide observations of CO_2_ exchange between terrestrial ecosystems and the atmosphere, using the eddy covariance method.^[^ ^54^ ^]^ Here, half‐hourly ER and soil moisture measurements from the FLUXNET2015 dataset were compiled to derive the ER‐soil moisture response curves. The night‐time ER estimates without gap fill were used (i.e., ER_NT_VUT_REF with QC = 0).^[^ ^55^ ^]^ Soil moisture was measured as volumetric soil water content (%). The surface soil moisture measurements (SM_1: 0 to 10 cm, varying across sites) were used, because deeper soil moisture measurements were only available at a very limited number of sites (less than 30).^[^ ^56^ ^]^ Sites without soil moisture measurements were removed, as were all wetland, snow and ice sites due to that they have a perched water table or are infrequently water‐limited.^[^ ^57^ ^]^ There are a total of 135 individual sites finally used in our study. Based on the International Geosphere‐Biosphere Programme (IGBP), these sites can be divided into 10 vegetation types: croplands (CRO), closed shrublands (CSH), deciduous broadleaf forests (DBF), evergreen broadleaf forests (EBF), evergreen needle‐leaf forests (ENF), grasslands (GRA), mixed forests (MF), open shrublands (OSH), savanna (SAV), and woody savanna (WSA).

### ER‐Soil Moisture Response Curves

Given that ecosystem responses to water variation may differ between growing and non‐growing seasons, this study focused only on deriving the ER‐soil moisture response curves during growing season, which was defined based on gross primary productivity (GPP; see Supporting Information). Following the basic concept of previously developed methods,^[^ ^44^, ^58^, ^59^, ^60^ ^]^ the soil moisture and its corresponding ER data for each site were first grouped into multiple bins by interval of 0.05%. Other intervals, such as 0.01% and 0.1%, were also tested, and the results were comparable to those using 0.05%. The 90% quantile of the ER was used as the response of ER within each soil moisture bin to minimize the possible influences of other limited factors.^[^ ^44^, ^59^ ^]^ Other quantiles, such as 85% and 95% were also tested, and the results were comparable to those using 90%. This was similar to the boundary line methodology and has been widely used in previous studies that relate biological responses to environmental factors.^[^ ^60^, ^61^, ^62^, ^63^ ^]^ The running averages of every three soil moisture bins were then calculated to construct the scatterplots of ER versus soil moisture,^[^ ^44^, ^64^ ^]^ and used generalized additive model (GAM) with the R package mgcv to fit the data. The running averages aim to make the data smoother.^[^ ^58^, ^65^ ^]^ The GAM was a nonparametric extension of generalized linear models, allows the data itself to determine the shape of the response curves, and thus eliminates the potential bias due to the choice of a specific function.^[^ ^66^, ^67^, ^68^ ^]^ Before performing these analyses, data outside the mean ± 2 times the standard deviation were excluded as outliers,^[^ ^69^, ^70^ ^]^ with aim of avoiding the impact of extreme values when deriving the ER‐soil moisture response curves.

### Estimation of Site‐Specific SMER opt

This study determined SMER opt based on the ER‐soil moisture response curves fitted by GAM, along with linear regression. In particular, sites identified with a unique soil moisture at which ER reached its maximum along the response curve were considered for the detection of SMER opt. The detection was successful only when the linear regression demonstrates increasing and declining trends in ER with increasing soil moisture below and above the SMER opt respectively (i.e., the red and blue segments shown in Figure 1). Among the 135 sites, the existence of SMER opt was successfully detected at 106 sites covering large areas and most vegetation types (Figure 2a; Table S1, Supporting Information). A site from each vegetation type was selected to exhibit a representative response curve (Figure 2b).

Considering that heat and light are potentially correlated or co‐vary with soil moisture,^[^ ^46^ ^]^ the robustness of the observed existence of SMER opt was tested against the impacts of incoming shortwave radiation, vapor pressure deficit, and air temperature, using both explainable machine learning and partial correlation‐based analysis. For the explainable machine learning, a random forest model using the R package randomForest was developed with ER as dependent variable and soil moisture, incoming shortwave radiation, vapor pressure deficit, and air temperature as independent variables. The SHAP (SHapley Additive exPlanations) values for soil moisture were then calculated with the R package treeshap, representing the response of ER to soil moisture after accounting for incoming shortwave radiation, vapor pressure deficit, and air temperature.^[^ ^46^ ^]^ Based on the SHAP values, the SMER opt was determined using the same method as used for the observational data aforementioned, which produced similar results (Figures S3 and S4, Supporting Information). In particular, the SHAP values‐derived SMER opt still existed at most sites, showing a close correlation with the observational data‐derived SMER opt. Thus, the observed widespread SMER opt was not likely to be caused by these confounding factors. Consistently, this was also supported by the partial correlation‐based analysis. It showed a strong linear relationship between the Spearman's correlation coefficients of soil moisture and ER without and with controlling for incoming shortwave radiation, vapor pressure deficit and air temperature (Figure S2, Supporting Information), suggesting that these factors had a negligible effect on the non‐linear relationship between soil moisture and ER and consequently the derived SMER opt.

### Drivers of the Distribution of SMER opt Across Sites

A serial of climatic, vegetation, and edaphic factors potentially influencing the spatial variability of SMER opt were explored. The climatic factors included mean air temperature (Tem,°C), incoming shortwave radiation (ISR, W m^−2^), vapor pressure deficit (VPD, kPa), soil moisture (SM_growth_, %), and soil temperature (ST,°C) during the growing season, as well as annual total precipitation (Pre, mm) from FLUXNET2015 dataset.^[^ ^54^ ^]^ For vegetation structure, the leaf areas index (LAI) for each site was extracted from global monthly LAI dataset according to its latitude and longitude.^[^ ^71^ ^]^ All the above variables were averaged for each site over the years of observation. The edaphic variables included soil bulk density (BD, kg dm^−3^), soil organic carbon (SOC, %), soil cation exchange capacity (CEC, cmol kg ^−1^), soil pH (pH) and soil sand fraction (Sand, %), which were directly retrieved from the Regridded Harmonized World Soil Database v.1.2 in the Oak Ridge National Laboratory Distributed Active Archive Center for Biogeochemical Dynamics (https://daac.ornl.gov/SOILS/guides/HWSD.html).

Given the possible collinearity among these climatic, vegetation, and edaphic factors, we used relative weight analysis (RWA) with the R package rwa to determine the relative importance of each variable on SMER opt. The RWA considers correlation among predictors, and partitions the explained variance across predictors by transforming correlated predictors into orthogonal variables, conducting a linear model on the transformed variables, and then transforming the resulting coefficients back to the original metric.^[^ ^72^, ^73^ ^]^ Ridge regression with the R package ridge was also conducted to quantify the independent effects of these factors on SMER opt.

### Statistical Analysis

R software version 4.3.0 was used to analyze and illustrate the data.^[^ ^74^ ^]^ Statistically significant differences were set with values of *p* ≤ 0.05.

## Conflict of Interest

The authors declare no conflict of interest.

## Author Contributions

S.N. supervised the study and wrote the manuscript draft. J.P. collected and analyzed the data, created graphs and tables, wrote the manuscript draft. S.X., J.T., J.L., C.C., C.S., Y.W., Q.Z., and G.Y. contributed to data analysis, interpretation, and wording. C.C. and J.L. conducted the experiment and collected the data. All authors revised the manuscript.

## Supporting information



Supporting Information

## Data Availability

FLUXNET data are downloaded from https://fluxnet.fluxdata.org. Leaf area index data are retrieved from https://daac.ornl.gov/cgi‐bin/dsviewer.pl?ds_id=1653. Soil properties are obtained from the Regridded Harmonized World Soil Database v.1.2 in the Oak Ridge National Laboratory Distributed Active Archive Center for Biogeochemical Dynamics (https://daac.ornl.gov/SOILS/guides/HWSD.html). Experimental data generated in this study can be accessed at https://doi.org/10.5281/zenodo.17376773.
