# Supplementary material for: Global Evidence of the Unimodal Response of Ecosystem Respiration to Soil Moisture
Source: Adv Sci (Weinh). 2025 Oct 30;13(3):e09753. doi: 10.1002/advs.202509753 (PMC12806489; doi:10.1002/advs.202509753)
Supplement: Supplementary file 1 — Supporting Information [file ADVS-13-e09753-s001.docx]

**Supplementary Information**

**Global Evidence of the Unimodal Response of Ecosystem Respiration to Soil Moisture**

*Jinlong Peng^1,2^, Shudi Xie^1,2^, Jiwang Tang^1,2^, Jiaqiang Liao^1,2^, Chen Chen^1,2^, Chuanlian Sun^2,3^, Yiheng Wang**^1,2^, Qingping Zhou^4^, Guirui Yu^1,2^, and Shuli Niu^1,2^**

*^1^Key Laboratory of Ecosystem Network Observation and Modeling,* *Institute of Geographic Sciences and Natural Resources Research, Chinese Academy of Sciences, Beijing 100101, China*

*^2^College of Resources and Environment, University of Chinese Academy of Sciences, Beijing 100049, China*

*^3^State Key Laboratory of Urban and Regional Ecology, Research Center for Eco-Environmental Sciences, Chinese Academy of Sciences, Beijing 100085, China*

*^4^Institute of Qinghai-Tibetan Plateau, Southwest Minzu University, Chengdu 610041, China*

**Corresponding author: Shuli Niu*

*Synthesis Research Center of Chinese Ecosystem Research Network, Key Laboratory of Ecosystem Network Observation and Modeling, Institute of Geographic Sciences and Natural Resources Research, Chinese Academy of Sciences, Beijing 100101 China.*

*Phone: 86-10-6488-8062*

*Fax: 86-10-6488-9399*

*Email: sniu@igsnrr.ac.cn*

**Supplementary Methods**

**Definition of growing season based on GPP**

GPP was used to determine the growing season at each FLUXNET stie. This is because GPP directly reflects the supply of respiratory substrates, as well as the temporal-spatial resolution of GPP observations is equivalent to that of ER observations in the FLUXNET dataset. The start (SOS) and end (EOS) dates of the growing season was determined by a dynamic phenological extraction threshold, which has been commonly used in previous studies.^[1,2]^ The determination of dynamic threshold is based on a simple linear function of the minimal (GPP_min_) and maximal (GPP_max_) values of GPP, i.e., GPP_0_ = GPP_min_ + 15% × (GPP_max_ - GPP_min_), where GPP_0_ represents the dynamic threshold of GPP used to identify SOS and EOS. SOS is defined as the day when the GPP seasonal curve crosses GPP_0_ within the GPP-up segment, while EOS is defined as the day when the GPP seasonal curve crosses GPP_0_ within the GPP-down segment. Thus, the length of the growing season is the difference between SOS and EOS.

**Calculation of ER sensitivity to soil moisture**

For each site with detected SMER opt, we estimated the ER-soil moisture linear slopes on both sides of the SMER opt by linear regression. The absolute slope values were used to represent ER sensitivity to soil moisture below (Sen_below_) and above SMER opt (Sen_above_), corresponding to the red and blue segments shown in Figure 1 respectively. Thus, a lager Sen_below_ indicates larger ER loss due to decreased soil moisture below SMER opt (i.e., soil moisture deficit), and a lager Sen_above_ indicates larger ER loss due to increased soil moisture above SMER opt (i.e., soil moisture excess). Then, we used paired Wilcoxon test with the R package stats to compare the difference between Sen_below_ and Sen_above_ across sites, which is robust against outliers and does not require the data to follow a specific distribution.^[3]^

**Experimental site and design**

The precipitation gradient experiment was conducted in an alpine meadow in Hongyuan County (32°50′ N, 102°34′ E, 3500 m a.s.l.) in the eastern part of the Qinghai-Tibetan Plateau. The site exhibits a typical continental plateau cold temperate monsoon climate, and the mean annual air temperature and precipitation were 1.5 ℃ and 747 mm respectively.^[4]^ The soil was classified as Mat Cry-gelic Cambisol.^[5]^ Within this field, the plant species can be divided into four functional types, that is *Anemone rivularis*-dominant forb type, *Deschampsia caespitosa*-dominant grass type, *Kobresia setchwanensis* and *Carex schneideri*-co-dominant sedge type, and *Oxytropis kansuensis*-dominant legume type.

This experiment began in 2015, and consists of six precipitation levels: 1/12P, 1/4P, 1/2P, 3/4P, P and 5/4P, where P was the annual precipitation. There were five replications under each precipitation level, thus 30 plots (each 2 × 3 m) in total, following a fully factorial randomized block design. The distance between each plot was 2 m. We installed rain interception shelters to achieve the different levels of precipitation. The rain interception shelters were made of acrylic transparent plastic, which could intercept different amounts of rainfall and minimally change other environmental variables, such as sunlight.^[6]^ Water catchments were used to estimate rainfall collected by each plot. We surrounded the plots with fiberglass plates installed to a depth of 40 cm to avoid lateral runoff.^[7]^ More detailed information on experimental site and design can be found in He et al.^[8]^

**Parameter measurements**

We conducted systematic measurements of ecosystem characteristics in 2022, during which precipitation treatments have significantly altered ecosystem structure and functioning as shown in He et al.^[8]^ For example, there were significant changes in soil moisture, species richness and dominance, and community stability along the precipitation gradient. These, in turn, would influence ecosystem water use capacity and potentially shift the response of ER to soil moisture,^[8]^ facilitating the capture of changes in the SMER opt under different precipitation treatments.

**ER and soil moisture**

ER was measured by a transparent static chamber (0.5 × 0.5 × 0.5 m) attached to an infrared gas analyzer (LI-6400, LI-COR, Lincoln, NE, USA). During measurements, the chamber was covered with an opaque cloth and was positioned over a square steel frame, which was permanently inserted into soil and offered a flat base for the chamber. Two electric fans were running continuously to mix air inside the chamber. Nine consecutive recordings of CO_2_ concentrations were taken at 10 s intervals after the gas concentration was linearly increasing within the chamber. We calculated ER as the rate of CO_2_ concentration change over time. ER measurements were conducted twice per month on cloudless days from June to August. More detailed information on measurement methods can be found in Niu et al.^[9]^ Simultaneous with each measurement of ER, soil moisture at a 10 cm depth in each plot was recorded using portable time domain reflectometry equipment (TDR 100, Spectrum Technologies Inc., Chicago, USA).

**Above- and** **belowground** **net primary production**

When plant biomass peaked in August, aboveground net primary production (ANPP) and belowground net primary production (BNPP) were measured. During measurement, in a 0.5 × 0.5 m quadrat that was chosen randomly within each plot, we harvested all living plants at the ground level. All plants were dried at 65 °C in an oven until constant weight to determine the ANPP. BNPP was measured using the ingrowth core method.^[10,11]^ More than 90% of the plant roots were distributed in the top 20 cm of the soil at this site, while almost 100% was distributed in the top 40 cm.^[11,12]^ To this end, we took soil cores (40 cm in depth, 9.0 cm in diameter) with two soil layers (0-20 cm, 20-40 cm) from the permanent position in each plot. These cores were refilled immediately with sieved root-free soil from the place outside of the plots that had identical soil properties as the sampling plot. We collected soil cores at the center of the original root ingrowth holes applying a smaller soil auger (7.4 cm in diameter). Root samples were obtained by washing these soil cores of different depths with a filter (0.25 mm) under slowly flowing water, then dried at 65 °C in an oven until constant weight to determine the BNPP. ANPP and BNPP were converted to g m^-2^.^[13]^

**Soil total carbon, total nitrogen, pH and inorganic N, and** **microbial biomass carbon and nitrogen**

After completing plant sampling, we collected soil samples at a depth of 0-10 cm in late August. Five soil cores were randomly collected using a soil auger (6 cm in diameter) and then mixed to form one composite sample. Each composite sample was divided into two parts after removing any visible plant materials and sifting through a 2 mm mesh sieve. One part was air-dried and used for measuring soil total carbon (TC) and nitrogen (TN) and soil pH, and other part was used for analyzing the soil inorganic N content (including NH_4_^+^ and NO_3_^-^) and microbial biomass carbon and nitrogen (MBC and MBN). Before measuring the TC and TN by an elemental analyzer (Vario EL III; Elementar, Germany), all soil samples were ground finely by a ball mill. The soil pH was determined with a glass electrode in a soil:water solution (1:2.5, w/v). Soil NH_4_^+^ and NO_3_^-^ were extracted using a 2 mol/L KCl solution and then measured by an automatic continuous flow analyzer (AA3, SEAL Analytical GmbH, Germany). The MBC and MBN were extracted and measured using chloroform direct-fumigation.^[14]^

**Statistical analysis for experimental data**

We derived the SMER opt in each precipitation level using the same method as used for the eddy covariance observations (see Method). We then averaged soil moisture within each precipitation level to represent growth soil moisture (SM_growth_), and used linear regression to test the relationship between SM_growth_ and SMER opt across precipitation levels. Finally, we used Pearson’s correlation analysis to quantify the role of a serial of plant, microbial, and soil properties (ANPP, BNPP, MBC, MBN, TC, TN, and soil NH_4_^+^, NO_3_^-^ and pH) in regulating SMER opt variation along SM_growth_. Statistically significant differences were set with values of *P* ≤ 0.05. R software version 4.3.0 was used to analyze and illustrate the data.^[15]^

**Supplementary** **Results**

**Influence of SM_growth_ on SMER opt in precipitation manipulation experiment**

To verify the causal effect of SM_growth_ on SMER opt, we derived the ER-soil moisture response curves and SMER opt at different precipitation levels in a field manipulation experiment (Figure S7). Among the six precipitation levels, we successfully detected the existence of SMER opt at 1/12P, 1/2P, 1/4P, and P. Further analysis showed that SMER opt exhibited a significant increasing trend over increasing SM_growth_ across these four precipitation levels, consistent with the eddy covariance results. Meanwhile, a rise in SM_growth_ significantly increased aboveground net primary productivity (ANPP) and microbial biomass carbon (MBC) (Figure S8). The two parameter was significantly correlated with SMER opt (Figure S9), suggesting that SM_growth_ shifted SMER opt mainly via changing ANPP and MBC along the precipitation gradient in this experiment.

**Sites without detected SMER opt in FLUXNET data**

For the 29 sites where SMER opt was absent, we further analyzed their ER-soil moisture response curves, and found that the absence of SMER opt was mainly due to two reasons. First, the soil moisture had wholly exceeded SMER opt with ER following a monotonic dynamic of decrease in response to rising soil moisture, making the detected SMER opt bias and unmeaningful. Second, in the range of soil moisture, the ER indeed had not reached optimum soil moisture without a clear peak in ER shown up. That being said, we cannot assume that these sites would have no ER peaks forever over time, and thus further investigation is needed when longer-term observations become available.

Data from 2 representative sites (CA-TP3 and US-SRC) supporting the above two reasons are plotted (Figure S10).


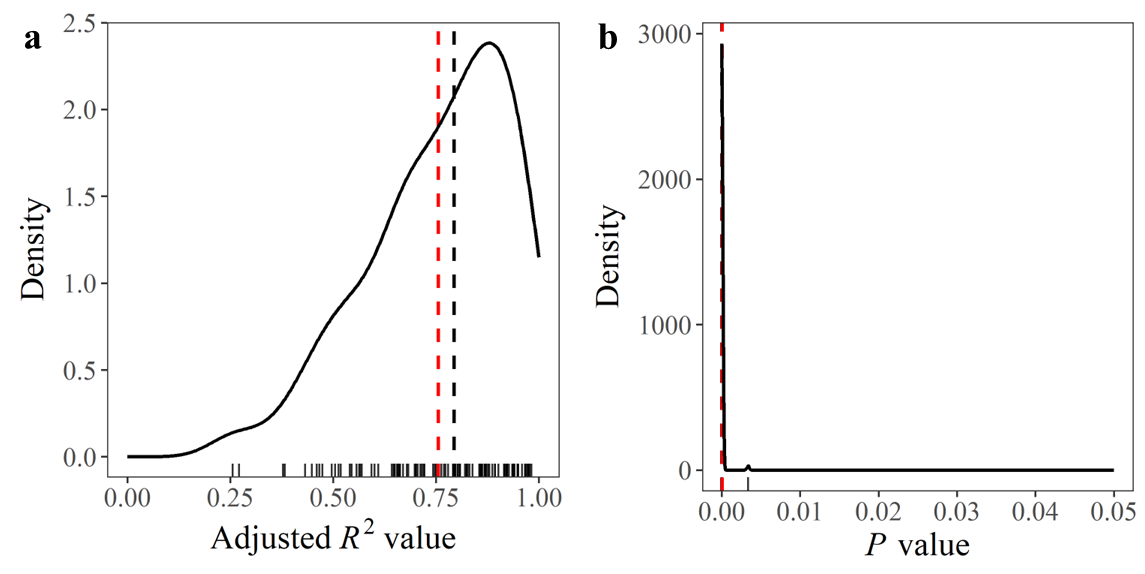


**Figure S1. Goodness-of-fit of the fitted generalized additive model (GAM) at the 106 sites with optimum soil moisture for ecosystem respiration (SMER opt).** **a** Distribution of adjusted *R*^2^ of model. **b** Distribution of *P* value of model. The black and red dotted lines are the median and mean respectively.


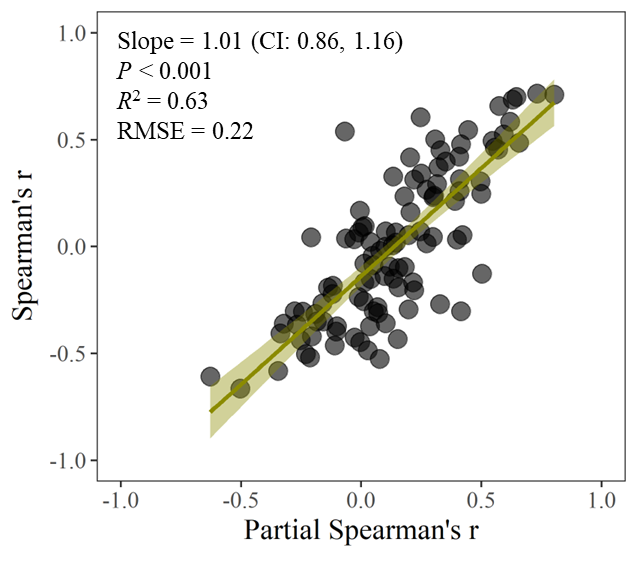


**Figure S2. Linear regression between the Spearman correlation coefficients of soil moisture and ecosystem respiration without and with controlling for air temperature, vapor pressure deficit, and incoming shortwave radiation (Spearman’s r and partial Spearman’s r).** Solid line and shaded area indicate the linear regression fit and its 95% confidence interval respectively. The CI represents the 95% confidence interval of linear regression slope, and the RMSE represents the root mean square error.

**
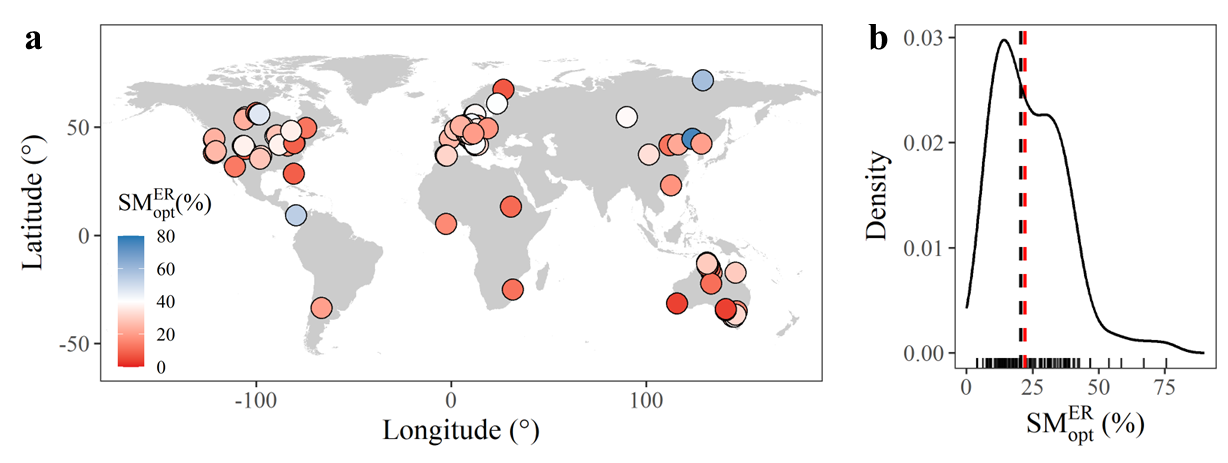
**

**Figure S3. Distribution of** **the SHAP values‐derived optimum soil moisture for ecosystem respiration (SMER opt).** **a** Location of the 103 sites with detected SMER opt. **b** Distribution of SMER opt values. The black and red dotted lines are the median and mean respectively.


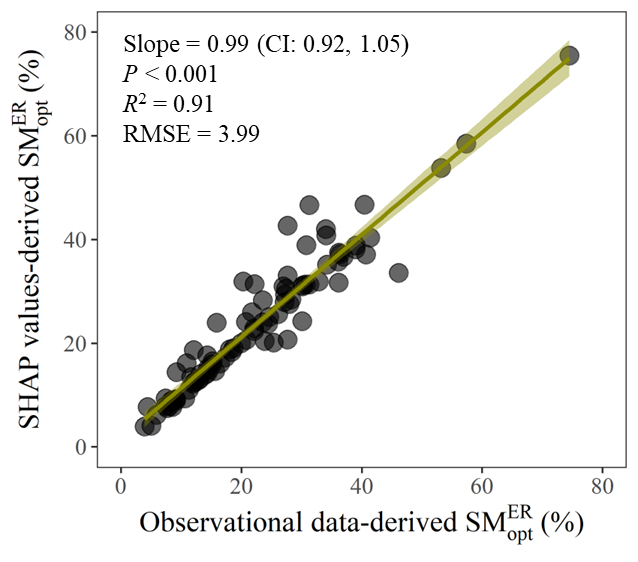


**Figure S4. Linear regression between SHAP values‐ and observational data‐derived optimum soil moisture for ecosystem respiration (SMER opt).** Solid line and shaded area indicate the linear regression fit and its 95% confidence interval respectively. The CI represents the 95% confidence interval of linear regression slope, and the RMSE represents the root mean square error.


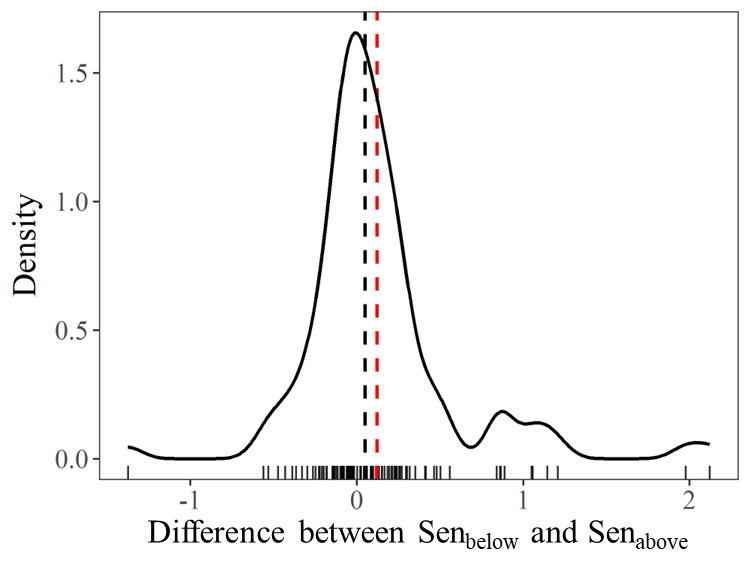


**Figure S5. Distribution of difference between ecosystem respiration sensitivity to soil moisture below (Sen_below_) and above SMER opt (Sen_above_).** The black and red dotted lines are the median and mean respectively.


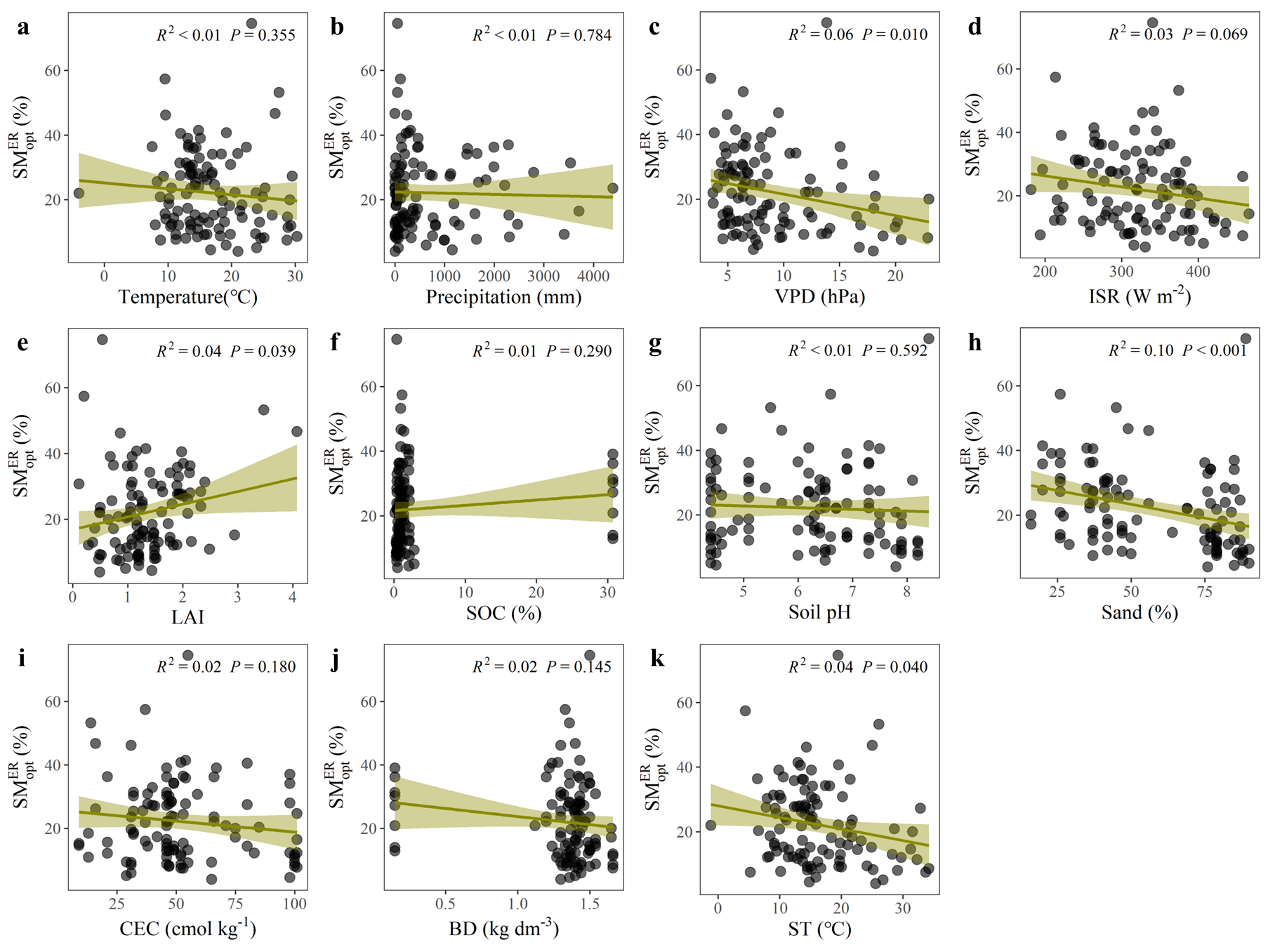


**Figure S6. Bivariate plots between optimum soil moisture for ecosystem respiration (SMER opt) and influencing variables.** The influencing variables are as follows: **a** air temperature; **b** total precipitation; **c** vapor pressure deficit; **d** incoming shortwave radiation; **e** leaf area index; **f** soil organic carbon; **g** soil pH; **h** soil sand fraction; **i** soil cation exchange capacity; **j** soil bulk density; **k** soil temperature. Solid line and shaded area indicate the linear regression fit and its 95% confidence interval respectively.


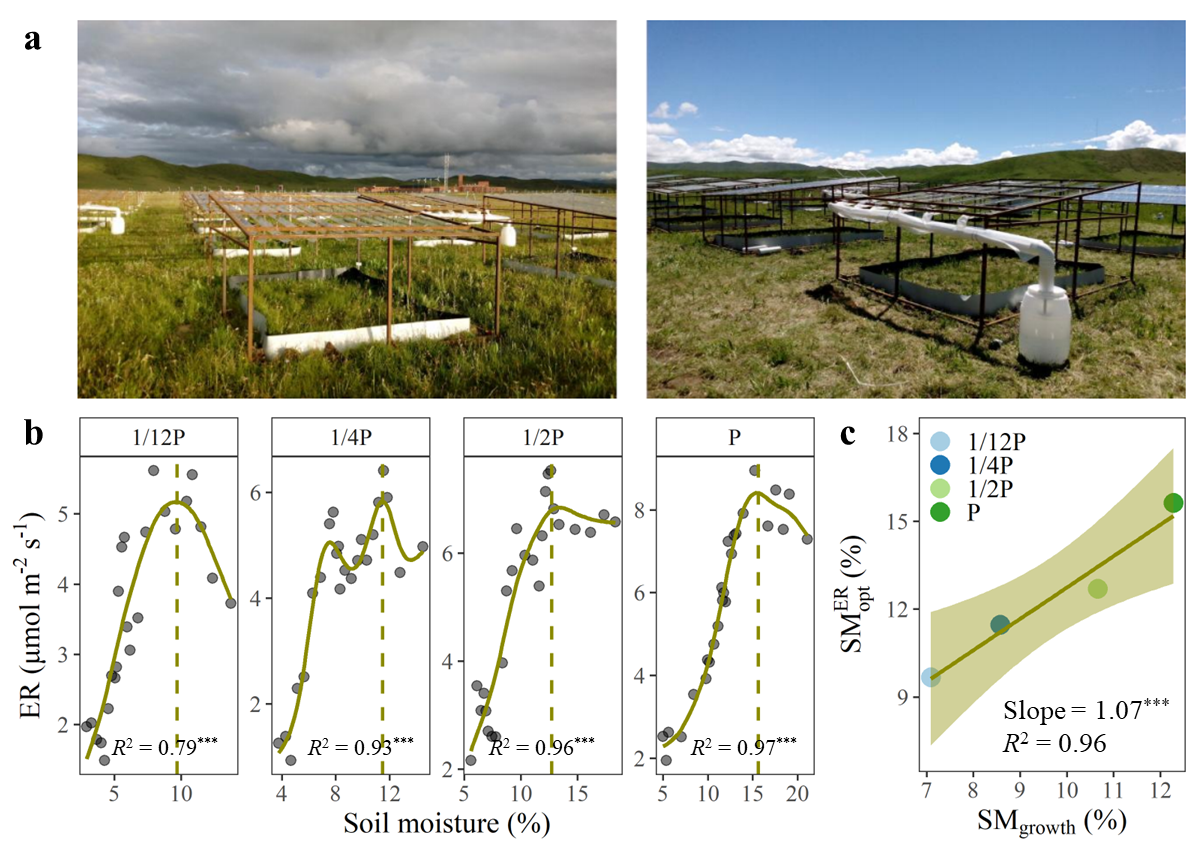


**Figure S7. Effect of growth soil moisture (SM_growth_) on** **optimum soil moisture for ecosystem respiration (SMER opt) derived from a field experiment manipulating precipitation.** **a** View of the experimental plots. **b** Response of ecosystem respiration (ER) to soil moisture in differential precipitation levels (1/12P, 1/4P, 1/2P, and P, where P was the annual precipitation). The solid curve indicates the fitting of generalized additive model (GAM), and the dotted line represents the detected SMER opt. **c** Linear regression of SM_growth_ to SMER opt across precipitation levels. Solid line and shaded area indicate the linear regression fit and its 95% confidence interval respectively. ***, *P* ≤ 0.001; **, *P* ≤ 0.01; *, *P* ≤ 0.05.

**
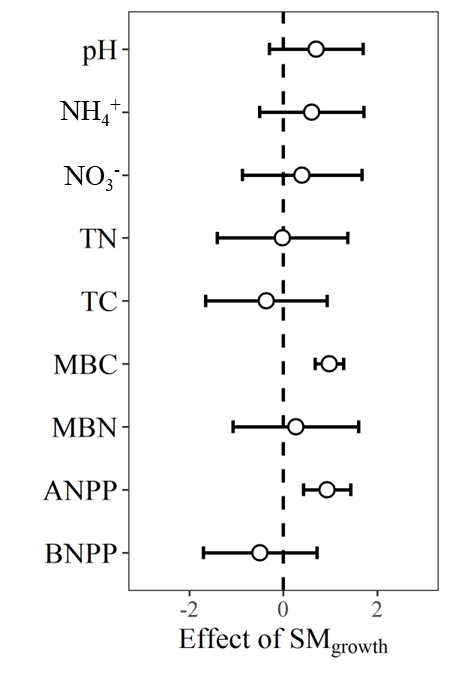
**

**Figure S8. Effects of growth soil moisture (SM_growth_) on plant, microbial, and soil properties derived from a field experiment manipulating precipitation.** The plant, microbial, and soil properties are as follows: pH, soil pH; NH_4_^+^, soil NH_4_^+^ (mg kg^-1^); NO_3_^-^, soil NO_3_^-^ (mg kg^-1^); TN, soil total nitrogen (%); TC, soil total carbon (%); MBC, microbial biomass carbon (mg kg^-1^); MBN, microbial biomass nitrogen (mg kg^-1^); ANPP, aboveground net primary production (g m^-2^); BNPP, belowground net primary production (g m^-2^). The circles and bars represent standardized regression slopes and 95% confidence intervals (CI) respectively, and the standardized regression slope was significant when the 95% CI did not overlap with zero (black vertical dotted line).


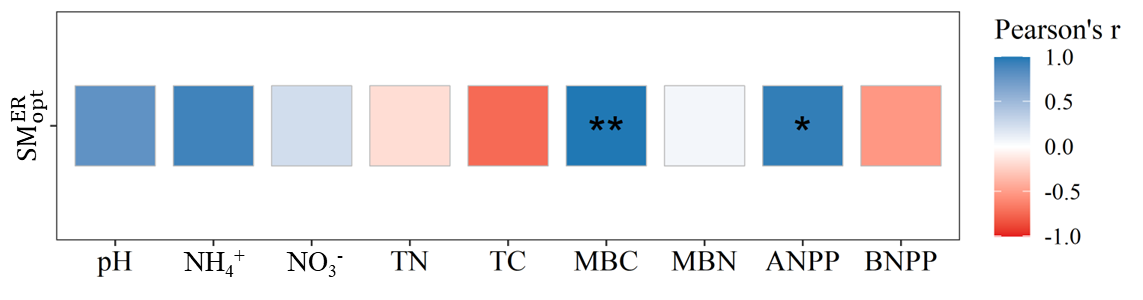


**Figure S9. Correlations between the optimum soil moisture for ecosystem respiration (SMER opt) and the plant, microbial, and soil properties derived from a field experiment manipulating precipitation.** pH, soil pH; NH_4_^+^, soil NH_4_^+^ (mg kg^-1^); NO_3_^-^, soil NO_3_^-^ (mg kg^-1^); TN, soil total nitrogen (%); TC, soil total carbon (%); MBC, microbial biomass carbon (mg kg^-1^); MBN, microbial biomass nitrogen (mg kg^-1^); ANPP, aboveground net primary production (g m^-2^); BNPP, belowground net primary production (g m^-2^). ***, *P* ≤ 0.001; **, *P* ≤ 0.01; *, *P* ≤ 0.05.


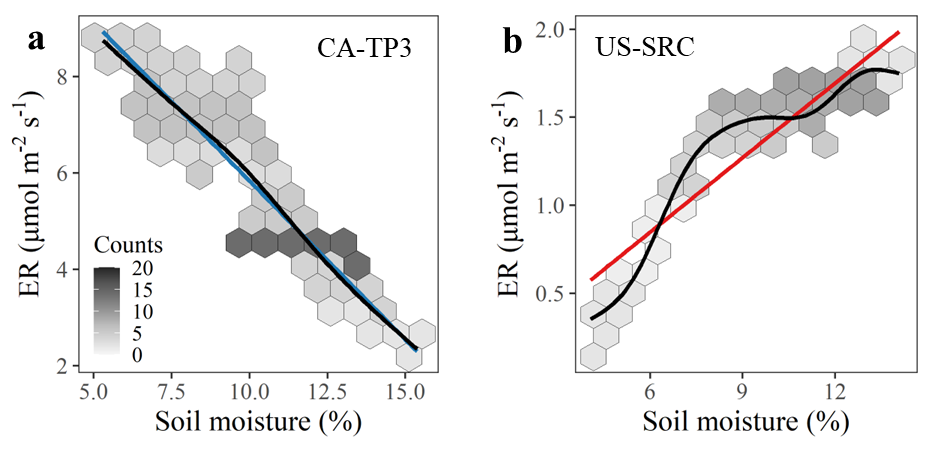


**Figure S10. Density plots showing the response of ecosystem respiration (ER) to soil moisture at two representative sites without detected optimum soil moisture for ER (SMER opt).** The black solid curve indicates the fitting of generalized additive model (GAM), and the colored line indicates the fitting of linear regression.

**Table S1 Information of 106 sites with** **detected** **optimum soil moisture for ecosystem respiration (SMER opt) in this study. MAP represents mean annual precipitation at each site.**

| Site name | Latitude | Longitude | MAP  (mm) | Period | IGBP |
| --- | --- | --- | --- | --- | --- |
| AR-SLu | -33.4648 | -66.4598 | 361.0 | 2009-2011 | MF |
| AT-Neu | 47.11667 | 11.3175 | 668.6 | 2002-2012 | GRA |
| AU-Ade | -13.0769 | 131.1178 | 1359.5 | 2007-2009 | WSA |
| AU-Cpr | -34.0021 | 140.5891 | 336.6 | 2010-2014 | SAV |
| AU-DaP | -14.0633 | 131.3181 | 1373.3 | 2007-2013 | GRA |
| AU-DaS | -14.1593 | 131.3881 | 1451.7 | 2008-2014 | SAV |
| AU-Dry | -15.2588 | 132.3706 | 920.3 | 2008-2014 | SAV |
| AU-Emr | -23.8587 | 148.4746 | 660.5 | 2011-2013 | GRA |
| AU-Gin | -31.3764 | 115.7138 | 620.1 | 2011-2014 | WSA |
| AU-How | -12.4943 | 131.1523 | 1715.4 | 2001-2014 | WSA |
| AU-Lox | -34.4704 | 140.6551 | 161.1 | 2008-2009 | DBF |
| AU-RDF | -14.5636 | 132.4776 | 1430.3 | 2011-2013 | WSA |
| AU-Rig | -36.6499 | 145.5759 | 445.0 | 2011-2014 | GRA |
| AU-Stp | -17.1507 | 133.3502 | 728.5 | 2008-2014 | GRA |
| AU-Whr | -36.6732 | 145.0294 | 389.0 | 2011-2014 | EBF |
| AU-Wom | -37.4222 | 144.0944 | 955.6 | 2010-2014 | EBF |
| AU-Ync | -34.9893 | 146.2907 | 692.3 | 2012-2014 | GRA |
| BE-Lon | 50.55162 | 4.74623 | 766.3 | 2004-2014 | CRO |
| BE-Vie | 50.30493 | 5.99812 | 951.9 | 1996-2014 | MF |
| CA-Gro | 48.2167 | -82.1556 | 821.2 | 2003-2014 | MF |
| CA-NS1 | 55.87917 | -98.48389 | 284.1 | 2001-2005 | ENF |
| CA-NS2 | 55.90583 | -98.52472 | 321.1 | 2001-2005 | ENF |
| CA-NS3 | 55.91167 | -98.38222 | 197.3 | 2001-2005 | ENF |
| CA-NS4 | 55.91437 | -98.38065 | 274.7 | 2002-2005 | ENF |
| CA-NS5 | 55.86306 | -98.485 | 288.9 | 2001-2005 | ENF |
| CA-NS6 | 55.91667 | -98.96444 | 254.7 | 2001-2005 | OSH |
| CA-NS7 | 56.63583 | -99.94833 | 302.1 | 2002-2005 | OSH |
| CA-Oas | 53.62889 | -106.1978 | 475.6 | 1996-2010 | DBF |
| CA-Qfo | 49.6925 | -74.34206 | 945.9 | 2003-2010 | ENF |
| CA-SF1 | 54.48503 | -105.8176 | 447.8 | 2003-2006 | ENF |
| CA-SF3 | 54.09156 | -106.0053 | 353.5 | 2001-2006 | OSH |
| CA-TP1 | 42.66094 | -80.55952 | 1107.8 | 2002-2014 | ENF |
| CA-TP4 | 42.71016 | -80.35738 | 1107.8 | 2002-2014 | ENF |
| CA-TPD | 42.63533 | -80.55773 | 920.8 | 2012-2014 | DBF |
| CH-Dav | 46.81533 | 9.85591 | 841.7 | 1997-2014 | ENF |
| CH-Fru | 47.11583 | 8.53778 | 1295.2 | 2005-2014 | GRA |
| CH-Lae | 47.47833 | 8.36439 | 1175.2 | 2004-2014 | MF |
| CH-Oe1 | 47.28583 | 7.73194 | 1222.0 | 2002-2008 | GRA |
| CH-Oe2 | 47.28642 | 7.73375 | 2063.5 | 2004-2014 | CRO |
| CN-Cha | 42.4025 | 128.0958 | 466.1 | 2003-2005 | MF |
| CN-Cng | 44.5934 | 123.5092 | 289.2 | 2007-2010 | GRA |
| CN-Dan | 30.4978 | 91.0664 | 520.2 | 2004-2005 | GRA |
| CN-Din | 23.1733 | 112.5361 | 1373.5 | 2003-2005 | EBF |
| CN-Du2 | 42.0467 | 116.2836 | 331.6 | 2006-2008 | GRA |
| CN-Du3 | 42.0551 | 116.2809 | 502.1 | 2009-2010 | GRA |
| CN-Ha2 | 37.6086 | 101.3269 | 527.0 | 2003-2005 | SAV |
| CN-HaM | 37.37 | 101.18 | 595.1 | 2002-2004 | GRA |
| CN-Sw2 | 41.7902 | 111.8971 | 280.7 | 2010-2012 | GRA |
| CZ-BK1 | 49.50208 | 18.53688 | 1291.9 | 2004-2014 | ENF |
| CZ-BK2 | 49.49443 | 18.54285 | 1223.1 | 2004-2012 | GRA |
| DE-Hai | 51.07921 | 10.45217 | 761.5 | 2000-2012 | DBF |
| DE-Lnf | 51.32822 | 10.3678 | 622.5 | 2002-2012 | DBF |
| DE-Obe | 50.78666 | 13.72129 | 1046.4 | 2008-2014 | ENF |
| DE-Seh | 50.87062 | 6.44965 | 574.2 | 2007-2010 | CRO |
| DK-Sor | 55.48587 | 11.64464 | 848.9 | 1996-2014 | DBF |
| ES-Amo | 36.83361 | -2.25232 | 293.7 | 2007-2012 | OSH |
| ES-LgS | 37.09794 | -2.96583 | 515.8 | 2007-2009 | OSH |
| ES-LJu | 36.92659 | -2.75212 | 595.0 | 2004-2013 | OSH |
| FI-Hyy | 61.84741 | 24.29477 | 604.0 | 1996-2014 | ENF |
| FI-Jok | 60.8986 | 23.51345 | 622.0 | 2000-2003 | CRO |
| FI-Sod | 67.36239 | 26.63859 | 529.1 | 2001-2014 | ENF |
| FR-Gri | 48.84422 | 1.95191 | 597.3 | 2004-2014 | CRO |
| FR-LBr | 44.71711 | -0.7693 | 920.2 | 1996-2008 | ENF |
| GH-Ank | 5.26854 | -2.69421 | 1795.4 | 2011-2014 | EBF |
| IT-CA1 | 42.38041 | 12.02656 | 767.6 | 2011-2014 | DBF |
| IT-CA2 | 42.37722 | 12.02604 | 767.6 | 2011-2014 | CRO |
| IT-CA3 | 42.38 | 12.0222 | 708.6 | 2011-2014 | DBF |
| IT-Col | 41.84936 | 13.58814 | 1178.6 | 1996-2014 | DBF |
| IT-Cp2 | 41.70427 | 12.35729 | 852.1 | 2012-2014 | EBF |
| IT-Cpz | 41.70525 | 12.37611 | 815.9 | 1997-2009 | EBF |
| IT-Isp | 45.81264 | 8.63358 | 2001.1 | 2013-2014 | DBF |
| IT-Lav | 45.9562 | 11.28132 | 1283.4 | 2003-2014 | ENF |
| IT-MBo | 46.01468 | 11.04583 | 974.0 | 2003-2013 | GRA |
| IT-Noe | 40.60618 | 8.15169 | 570.7 | 2004-2014 | CSH |
| IT-PT1 | 45.20087 | 9.06104 | 781.7 | 2002-2004 | DBF |
| IT-Ren | 46.58686 | 11.43369 | 915.7 | 1998-2013 | ENF |
| IT-Ro1 | 42.40812 | 11.93001 | 811.0 | 2000-2008 | DBF |
| IT-SR2 | 43.73202 | 10.29091 | 1328.7 | 2013-2014 | ENF |
| IT-Tor | 45.84444 | 7.57806 | 767.8 | 2008-2014 | GRA |
| JP-SMF | 35.2617 | 137.0788 | 1534.0 | 2002-2006 | MF |
| MY-PSO | 2.973 | 102.3062 | 1864.8 | 2003-2009 | EBF |
| NL-Loo | 52.16658 | 5.74356 | 828.7 | 1996-2014 | ENF |
| PA-SPn | 9.31814 | -79.6346 | 2239.3 | 2007-2009 | DBF |
| RU-Ha1 | 54.72517 | 90.00215 | 383.7 | 2002-2004 | GRA |
| RU-Tks | 71.59427 | 128.88782 | 272.6 | 2010-2014 | GRA |
| US-ARb | 35.5497 | -98.0402 | 710.6 | 2005-2006 | GRA |
| US-ARc | 35.54649 | -98.04 | 762.8 | 2005-2006 | GRA |
| US-ARM | 36.6058 | -97.4888 | 645.9 | 2003-2012 | CRO |
| US-Blo | 38.8953 | -120.6328 | 1374.8 | 1997-2007 | ENF |
| US-GLE | 41.36653 | -106.2399 | 1418.6 | 2004-2014 | ENF |
| US-IB2 | 41.84062 | -88.24103 | 971.6 | 2004-2011 | GRA |
| US-KS2 | 28.6086 | -80.6715 | 1146.4 | 2003-2006 | CSH |
| US-Me2 | 44.4523 | -121.5574 | 486.0 | 2002-2014 | ENF |
| US-Me3 | 44.3154 | -121.6078 | 374.8 | 2004-2009 | ENF |
| US-Me5 | 44.43719 | -121.5668 | 402.4 | 2000-2002 | ENF |
| US-Me6 | 44.32328 | -121.6078 | 408.2 | 2010-2014 | ENF |
| US-NR1 | 40.0329 | -105.5464 | 721.3 | 1998-2014 | ENF |
| US-Oho | 41.5545 | -83.8438 | 834.4 | 2004-2013 | DBF |
| US-SRM | 31.8214 | -110.8661 | 333.2 | 2004-2014 | WSA |
| US-Syv | 46.242 | -89.3477 | 665.4 | 2001-2014 | MF |
| US-Ton | 38.4316 | -120.966 | 545.7 | 2001-2014 | WSA |
| US-UMd | 45.5625 | -84.6975 | 717.5 | 2007-2014 | DBF |
| US-Var | 38.4133 | -120.9507 | 572.3 | 2000-2014 | GRA |
| US-WCr | 45.8059 | -90.0799 | 693.1 | 1999-2014 | DBF |
| US-Wkg | 31.7365 | -109.9419 | 292.4 | 2004-2014 | GRA |
| ZM-Mon | -15.4391 | 23.2525 | 582.2 | 2000-2009 | DBF |

**Supplementary References**

1. Wu, W., Sun, Y., Xiao, K., & Xin, Q. C. (2021). Development of a global annual land surface phenology dataset for 1982-2018 from the AVHRR data by implementing multiple phenology retrieving methods. International Journal of Applied Earth Observation and Geoinformation, 103. doi:10.1016/j.jag.2021.102487
2. Wang, F. Y., Xue, M. M., Zhou, L. M., Doughty, C. E., Ciais, P., Reich, P. B., . . . Chen, X. Z. (2025). Contrasting age-dependent leaf acclimation strategies drive vegetation greening across deciduous broadleaf forests in mid- to high latitudes. Nature Plants, 11(9). doi:10.1038/s41477-025-02096-5
3. Hollander, M. & Wolfe, D. A. (1973). Nonparametric Statistical Methods. New York: John Wiley & Sons.
4. Quan, Q., Zhang, F. Y., Jiang, L., Chen, H. Y. H., Wang, J. S., Ma, F. F., Song, B., & Niu, S. L. (2021). High-level rather than low-level warming destabilizes plant community biomass production. Journal of Ecology, 109(4), 1607-1617. doi:10.1111/1365-2745.13583
5. Li, G. Y., & Sun, S. C. (2011). Plant clipping may cause overestimation of soil respiration in a Tibetan alpine meadow, southwest China. Ecological Research, 26(3), 497-504. doi:10.1007/s11284-011-0806-7
6. Yahdjian, L., & Sala, O. E. (2002). A rainout shelter design for intercepting different amounts of rainfall. Oecologia, 133(2), 95-101. doi:10.1007/s00442-002-1024-3
7. Zavaleta, E. S., Shaw, M. R., Chiariello, N. R., Thomas, B. D., Cleland, E. E., Field, C. B., & Mooney, H. A. (2003). Grassland responses to three years of elevated temperature, CO2, precipitation, and N deposition. Ecological Monographs, 73(4), 585-604. doi:10.1890/02-4053
8. He, Y. L., Wang, J. S., Tian, D. S., Quan, Q., Jiang, L., Ma, F. F., Yang, L., Zhang, F. Y., Zhou, Q. P., & Niu, S. L. (2022). Long-term drought aggravates instability of alpine grassland productivity to extreme climatic event. Ecology, 103(11). doi:10.1002/ecy.3792
9. Niu, S. L., Wu, M. Y., Han, Y., Xia, J. Y., Li, L. H., & Wan, S. Q. (2008). Water-mediated responses of ecosystem carbon fluxes to climatic change in a temperate steppe. New Phytologist, 177(1), 209-219. doi:10.1111/j.1469-8137.2007.02237.x
10. Wilcox, K. R., von Fischer, J. C., Muscha, J. M., Petersen, M. K., & Knapp, A. K. (2015). Contrasting above- and belowground sensitivity of three Great Plains grasslands to altered rainfall regimes. Global Change Biology, 21(1), 335-344. doi:10.1111/gcb.12673
11. Quan, Q., Zhang, F., Meng, C., Ma, F., Zhou, Q., Sun, F., & Niu, S. (2020). Shifting biomass allocation determines community water use efficiency under climate warming. Environmental Research Letters, 15(9). doi:10.1088/1748-9326/aba472
12. Ma, F., Chen, W., Wang, J., Tian, D., Zhou, Q., & Niu, S. (2023). Below-ground net primary productivity stability in response to a nitrogen addition gradient in an alpine meadow. Functional Ecology, 37(2), 315-326. doi:10.1111/1365-2435.14236
13. Seabloom, E. W., Borer, E. T., Hobbie, S. E., & MacDougall, A. S. (2021). Soil nutrients increase long-term soil carbon gains threefold on retired farmland. Global Change Biology, 00, 1–12. doi:10.1111/gcb.15778
14. Vance, E. D., Brookes, P. C., Jenkinson, D. S. (1987). An extraction method for measuringsoil microbial biomass C. Soil Biology and Biochemistry. 19, 703–707 doi:10.1016/0038-0717(87)90052-6
15. R Core Team. (2023). R: A language and environment for statistical computing. R Foundation for Statistical Computing. https://www.R-project.org/
